# Motivation and value influences in the relative balance of goal-directed and habitual behaviours in obsessive-compulsive disorder

**DOI:** 10.1038/tp.2015.165

**Published:** 2015-11-03

**Authors:** V Voon, K Baek, J Enander, Y Worbe, L S Morris, N A Harrison, T W Robbins, C Rück, N Daw

**Affiliations:** 1 Department of Psychiatry, Addenbrookes Hospital, University of Cambridge, Cambridge, UK; 2 Department of Psychology, Behavioural and Clinical Neuroscience Institute, University of Cambridge, Cambridge, UK; 3 Cambridgeshire and Peterborough NHS Foundation Trust, Cambridge, UK; 4 Division of Psychiatry, Department of Clinical Neuroscience, Karolinska Institute, Stockholm, Sweden; 5 Department of Psychology, University of Cambridge, Cambridge, UK; 6 Department of Psychiatry, Brighton and Sussex Medical School, University of Sussex, Brighton, UK; 7 Center for Neural Science, Department of Psychology, New York University, New York, NY, USA

## Abstract

Our decisions are based on parallel and competing systems of goal-directed and habitual learning, systems which can be impaired in pathological behaviours. Here we focus on the influence of motivation and compare reward and loss outcomes in subjects with obsessive-compulsive disorder (OCD) on model-based goal-directed and model-free habitual behaviours using the two-step task. We further investigate the relationship with acquisition learning using a one-step probabilistic learning task. Forty-eight OCD subjects and 96 healthy volunteers were tested on a reward and 30 OCD subjects and 53 healthy volunteers on the loss version of the two-step task. Thirty-six OCD subjects and 72 healthy volunteers were also tested on a one-step reversal task. OCD subjects compared with healthy volunteers were less goal oriented (model-based) and more habitual (model-free) to reward outcomes with a shift towards greater model-based and lower habitual choices to loss outcomes. OCD subjects also had enhanced acquisition learning to loss outcomes on the one-step task, which correlated with goal-directed learning in the two-step task. OCD subjects had greater stay behaviours or perseveration in the one-step task irrespective of outcome. Compulsion severity was correlated with habitual learning in the reward condition. Obsession severity was correlated with greater switching after loss outcomes. In healthy volunteers, we further show that greater reward magnitudes are associated with a shift towards greater goal-directed learning further emphasizing the role of outcome salience. Our results highlight an important influence of motivation on learning processes in OCD and suggest that distinct clinical strategies based on valence may be warranted.

## Introduction

Our decisions are based on parallel and competing systems of goal-directed and habitual learning. Goal-directed behaviours are based on a flexible tracking of affective outcomes and models of the environment, whereas habitual behaviours are automated efficient choices based on previously reinforced actions.^1, 2, 3^ These learning systems have also been termed model-based and model-free, respectively.^4^ Using a recently developed two-step sequential decision task, healthy volunteers (HVs) show parallel engagement of model-based goal-directed and model-free habitual processes.^5^ This relative balance can shift in disorders of compulsivity such as obsessive-compulsive disorder (OCD) or disorders of addiction characterized by a shift away from goal-directed towards habitual behaviours.^6^ Here we sought to investigate the role of valence (gain or loss) outcome on the relative balance of these systems of learning in OCD.

Evidence from studies using implicit learning tasks suggests that OCD subjects are aberrantly over-reliant on goal-directed neural systems.^7, 8, 9, 10^ In contrast, OCD has also been associated with impairments in goal-directed learning with impaired awareness of explicit stimulus-outcome contingencies.^11^ Impaired goal-directed and enhanced habit learning has also been demonstrated in a ‘slips of action' task with OCD subjects showing enhanced response to outcomes that have been devalued and are no longer favourable.^11^ Similarly, using a two-step task, we have shown that OCD is associated with a shift away from goal-directed towards habitual behaviours.^6^ We aim to reconcile the discussed findings and while we hypothesize that OCD subjects will be more habitual in the context of reward, we expect divergent effect of loss on the balance between goal-directed and habitual behaviours.

The discussed literature suggests that OCD subjects may be particularly sensitive to negative or aversive outcomes and indeed, the clinical phenotypes of checking and obsessional thoughts are significantly associated with harm avoidance.^12^ However, because many of the studies examining the role of habits versus goal-directed learning utilize rewarding or ‘correct' outcomes, the influence of motivational states or value remains to be examined. For instance, using a probabilistic selection task, OCD subjects were better and faster at avoiding stimuli associated with negative feedback than approaching stimuli associated with positive feedback.^13^ Aberrant neural responses to reward and loss have also been demonstrated; OCD subjects showed greater activity in medial and superior frontal cortex and anterior cingulate when anticipating loss and decreased activity when anticipating reward using the monetary incentive delay task.^14^ Decreased activity of the dorsal striatum to reward anticipation^15^ and to loss receipt have also been shown in OCD.^16^ Furthermore, OCD is associated with enhanced habit learning of shock avoidance,^17^ but how the relative balance of goal-directed versus habitual behaviours may be affected specifically by negative outcomes is not yet clear. Although habit expression is commonly assessed by the testing of responses to devalued outcomes following over-learning, the two-step task used in the current study tests the relative balance of goal-directed and habitual processes during the learning process itself.

In the first study, we test the influence of reward and loss outcomes on the relative balance of goal-directed and habitual behaviours in OCD using the two-step task. In a previous study, we had shown that reward outcomes alter the relative balance of learning systems, causing a shift towards habitual learning;^6^ here we extend these findings in a larger group of subjects and assess the separate influences of both learning systems on reward and loss outcomes. In the second study, we further compare these results with a one-step acquisition learning and reversal-learning task modified to assess the role of altered magnitudes of reward and loss outcomes. Reversal learning is the capacity to shift learned action–outcome responses following changes in action–outcome contingencies and implicates the orbitofrontal cortex. Despite differences in the orbitofrontal cortex volume and activity, OCD has not been consistently associated with impairments in reversal learning.^18^ Thus we hypothesize that OCD is associated with enhanced acquisition errors to loss outcomes.

## Materials and methods

### Recruitment

OCD subjects were recruited from community and university-based advertisements and self-help groups in the East Anglia region. Subjects were also recruited from a psychiatric clinic at the Karolinska Institutet, Stockholm, Sweden. The OCD diagnosis was confirmed by a psychiatrist (VV, CR) using the Diagnostic and Statistical Manual of Mental Disorders, Version IV (DSM IV-TR) criteria.^19^ Age- and gender-matched HVs were recruited via community and university-based advertisements in the East Anglia region or in Stockholm. OCD subjects were included if they had a Yale Brown Obsessive Compulsive Scale (YBOCS)^20^ score >11. Subjects >18 years old were included. A separate group of 15 HVs were recruited to test for order effects. Subjects were excluded if they had a current major depression or other major psychiatric disorder including substance addiction, or major medical or neurological illness. HVs were medication free. Subjects were screened for comorbid psychiatric disorders with the Mini International Neuropsychiatric Inventory. The National Adult Reading Test^21^ was used to obtain an index of premorbid IQ. All subjects also completed the Beck Depression Inventory (BDI).^22^ Subjects were paid for their study participation time and told they could receive an additional amount (£5) dependent on their performance. All the subjects provided informed consent. The study was approved by the University of Cambridge Research Ethics Committee and the regional ethical review board in Stockholm, Sweden.

#### Model-free model-based task

**Figure 1:**
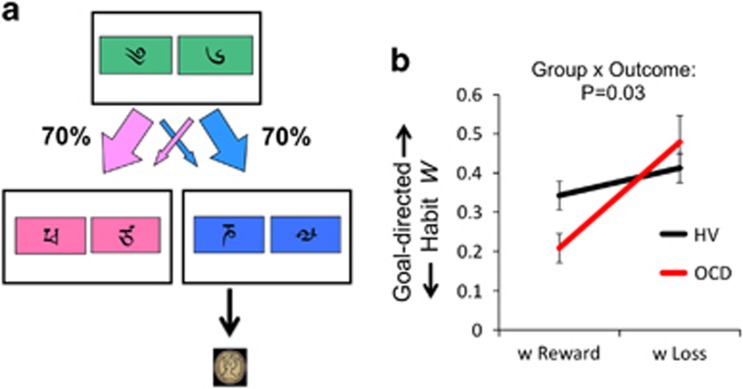
Two-step task. (**a**) Two-step task. (**b**) The graph shows *w* as a function of reward and loss outcome in obsessive-compulsive disorder (OCD) and healthy volunteer (HV) subjects. Group × outcome interaction: *P*=0.033. Error bars represent s.e.m.

Subjects underwent extensive computer-based instructions explaining concepts and providing practice examples of changes in transition and probability, and the two-stage task structure.^5^ Instructions were self-paced and lasted 15 to 20 min. Subjects chose between a stimulus pair at stage 1. The choice of a stimulus at stage 1 led with a fixed probability to one of two stimulus pairs at stage 2 (*P*=0.70 or 0.30) with the other stimulus leading to the two stimulus pairs with opposite probability (*P*=0.30 or 0.70). Choice of a stimulus at stage 2 led to a reward with probability varying slowly and independently over time (between *P*=0.25 to 0.75; Figure 1). Participants were overtly informed that a stimulus at stage 1 led to one of two stimulus pairs at stage 2 with a fixed probability. These probabilities were learned through experience during training. Four different reward probability distributions were used, which was counterbalanced in each group. Subjects were given 2 s to make a decision at each stage. The transition between stage 1 to stage 2 was 1.5 s. The stimulus chosen in stage 1 remained on the screen in stage 2 as a reminder. The stimulus chosen in stage 2 remained on the screen in the feedback stage as a reminder.

For the comparison of reward and loss learning, the OCD subjects and HVs were tested under two conditions of £1 reward followed by £1 loss with differing stimuli separated by at least 30 min between the two conditions. The loss condition was also preceded by its own training instructions. In the testing for an order effect in HVs, subjects were tested with either £1 reward or £1 loss in a counterbalanced order. If subjects started with the £1 loss condition, they were shown a £5 note indicating that they started with this amount and would lose a proportion of this dependent on their earnings. The outcomes were images of £1 or £1 with a red ‘X' across an image for subjects tested in the United Kingdom or 1 Kronor for subjects tested in Sweden. Subjects completed 201 trials divided into three sessions (7.5 s per trial, 8.38 min per session) per condition. The task was run using MATLAB 2011a.

#### Reversal learning

**Figure 2:**
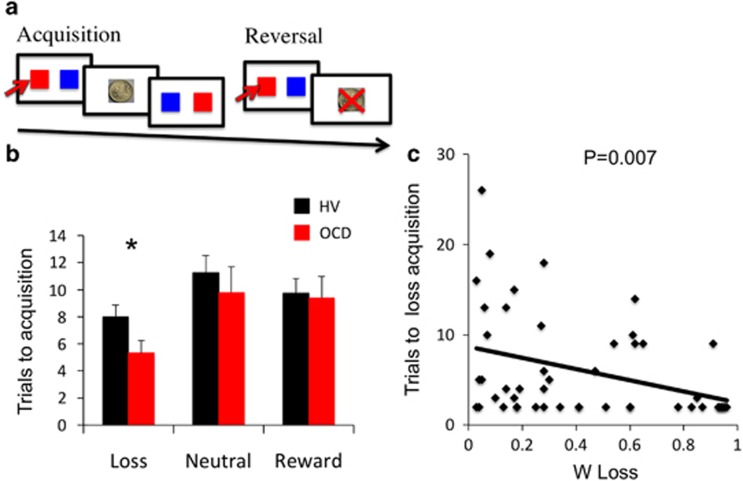
Acquisition and reversal learning one-step task. (**a**) Acquisition and reversal learning one-step task. (**b**) The bar graph shows the number of trials to acquisition for obsessive-compulsive disorder (OCD) and healthy volunteer (HV) subjects as a function of outcome. Error bars represent s.e.m. *P=0.005. (**c**) The regression plot shows the relationship between trials to acquisition for the loss condition of the one-step task and *w* score for the loss condition of the two-step task.

We used a modified one-step probabilistic reversal-learning task (Figure 2a). In the acquisition phase, subjects chose from one of three stimulus pairs probabilistically associated with outcomes varying by reward and loss outcome magnitude: loss (stimulus A: *P*=0.30 Win +£1/*P*=0.70 Lose −£2 (mean −£1.1); stimulus B: *P*=0.70 Win +£1/*P*=0.30 Lose −£2 (mean +£0.1)), neutral (stimulus C: *P*=0.70 Win +£1/*P*=0.30 Lose −£1 (mean+£0.4); stimulus D: *P*=0.30 Win +£1/*P*=0.70 Lose −£1 (mean −£0.4)) or reward (stimulus E: *P*=0.70 Win +£2/*P*=0.30 Lose −£1 (mean+£1,1); stimulus F: *P*=0.30 Win +£2/*P*=0.70 Lose −£1 (mean+0.1)). Following 30 trials per condition, contingencies for each stimulus pair switched being then followed by 30 trials per condition in the reversal phase (for example, stimulus A: *P*=0.70 Win +£1/*P*=0.3 Lose −£2; stimulus B: *P*=0.30 Win +£1/*P*=0.70 Lose −£2).

The stimulus phase was shown for 2.5 s during which the subjects indicated a response by pressing the left arrow on the keyboard for the stimulus on the left and the right arrow on the keyboard for the stimulus on the right. The stimuli were present on the screen until the subjects responded. If subjects were too slow, this was followed by the words: ‘You were too slow. Respond faster.' The stimulus phase was followed by a 1 s outcome phase with the words ‘You WON!!' and an image of a £2 or £1 coin or ‘You LOST!!' and an image of a large red ‘X' over the £2 or £1 coin shown for 1 s. The position of the stimuli within each stimulus pair was counterbalanced on either side of the screen and the stimuli conditions were randomly presented. The trial was followed by a variable intertrial interval of a mean of 0.75 s varying between 0.5 and 1 s. The primary outcome measure was the number of trials to criterion of four correct sequential choices. Other outcome measures included win-stay and lose-shift. The task was programmed in E-prime Version 2.

#### Data analysis

Two-step task: computational model (adapted from Daw *et al.*^5^).

The computational and behavioural analysis are described in Supplementary Materials.

### Statistics

Subject characteristics were compared using *t*-tests or Chi-square analysis. Data were inspected for outliers (>3 s.d. above group mean) and tested for normality of distribution using Shapiro–Wilks test. Data that were normally distributed were analysed using an independent *t*-test (for the reward condition with variance tested using Levene's test) or mixed-measures analysis of variance (ANOVA; for the reward and loss condition) to compare between groups with Pearson correlation used for correlation analyses. Data that were not normally distributed (Shapiro–Wilks: *P*>0.05) were indicated in the text and non-parametric Mann–Whitney *U*-tests were used to compare between groups and Spearman Rank test used for correlation analyses.

## Results

Subject characteristics are reported in Table 1 for subjects who completed both the reward and loss versions of the task. We also directly compared OCD subjects between the two sites: there were no differences in gender (Cambridge: male=4; KI: male=12, Chi-square=0.81, *P*=0.290), age (Cambridge: 41.67 (s.d. 13.83); KI: 34.21 (s.d. 12.89); *P*=0.100); IQ (Cambridge 114.49 (s.d. 6.64); KI: 117.11 (s.d. 5.70); *P*=0.263), BDI (Cambridge 16.03 (s.d. 10.59); KI: 13.98 (s.d. 8.88), *P*=0.491) or YBOCS (Cambridge 22.28 (s.d. 3.54); KI 20.20 (s.d. 6.19), *P*=0.256). We examined the weighting factor, *w*, calculated for each individual and describing the relative contribution of either habitual (model-free, *w*=0) or goal-directed (model-based, *w*=1) decision-making.

### Study 1: OCD: two-step task

#### Computational analysis

The reward condition in the OCD subjects have been previously described (*N*=30)^6^ and extended in sample size in this study (*N*=48: *N*=33 Cambridge; *N*=15 Karolinska Institutet; matched HVs: *N*=96 including *N*=15 HVs from Karolinska). We first assessed the effects of *w* on reward in this larger sample to confirm our previous findings. OCD subjects had lower *w* in the reward condition in this larger sample size compared with HVs (OCD: 0.22 (s.d. 0.20); HV: 0.33 (s.d. 0.25); *t*=2.72, *P*=0.008).

To assess our *a priori* hypothesis, we then compared the effects of reward and loss outcomes tested within subjects in OCD (*N*=30) and matched HVs (*N*=53) analysing *w* using a mixed-measures ANOVA with outcome as a within-subjects factor and group as a between-subjects factor. There was a main effect of outcome (F(1,81)=14.87, *P*<0.0001) and a group × outcome interaction (F(1,81)=4.71, *P*=0.033). Given this interaction, we assessed *post hoc* differences using Tukey test: OCD subjects had lower *w* (less goal-directed) scores to reward outcomes (*P*=0.013) but not to loss outcomes (*P*=0.385) compared with HVs. OCD subjects (*P*<0.0001) also had lower *w* in reward compared with loss with no differences in HVs (*P*=0.165). There was no main effect of group (F(1,81)=0.819, *P*=0.368; Figure 1b). Other parameters are reported in Table 1.

**Figure 3:**
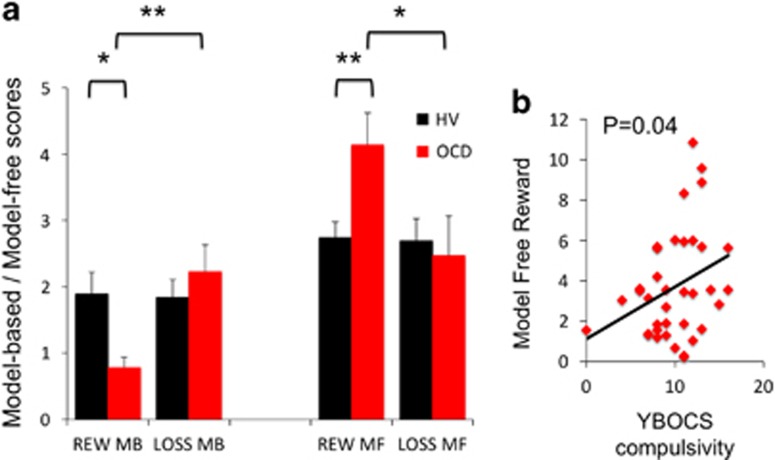
Model-free and model-based analyses of two-step task. (**a**) The graph shows the separate analyses of model-based (MB) goal-directed and model-free (MF) habitual scores for reward (REW) and loss (LOSS) outcomes in obsessive-compulsive disorder (OCD) and healthy volunteer (HV) subjects. Group × outcome × learning interaction: *P*=0.005. *P<0.05, **P<0.01. Error bars represent s.e.m. (**b**) The regression plot shows the relationship between MF reward outcomes and the Yale Brown Obsessive Compulsive Scale score for compulsive symptoms.

We then calculated computational model-based and model-free measures, which were further separable by computing model-based=β1 × *w* and model-free=β1 × (1−*w*), respectively and used a mixed-measures ANOVA comparing the between-subjects group factor (OCD, HV), and the within-subjects factor of outcome (reward, loss) and learning (model-based, model-free) measures (Figure 3). There was a main effect of learning (F(1,81)=34.23, *P*<0.0001), a group × learning interaction (F(1,81)=4.48, *P*=0.033) and a group × outcome × learning interaction (F(1,81)=8.49, *P*=0.005). There was no main effect of group (F(1,81)=0.207, *P*=0.650). Given the group × outcome × learning interaction, we then conducted *post hoc* analyses to further understand this interaction. OCD subjects had both lower model-based goal-directed learning (*P*=0.014) and greater model-free learning (*P*=0.005) to reward outcomes compared with HVs with no group differences to loss outcomes (*P*>0.05). In OCD subjects, model-based learning was lower to reward relative to loss outcomes (*P*=0.008) and model-free learning was higher to reward relative to loss outcomes (*P*=0.014), which was not observed in HV subjects (*P*>0.05).

On an exploratory basis, we then asked whether OCD severity was correlated with model-based or model-free scores. YBOCS compulsivity scores were positively correlated with model-free scores to reward (*R*=0.340, *P*=0.043; Figure 3) but not to model-based or loss outcomes. YBOCS obsessional scores were also not correlated with model-based or model-free scores.

We also asked whether there was a relationship with depression scores with reward and loss outcomes. We analysed *w* for reward and loss outcomes separately using univariate analyses with depression scores as a covariate. The difference between groups in the reward domain remained significant (*P*=0.02) and the loss domain was not significant (*P*=0.577). To further understand this on an exploratory basis, we asked whether *w* correlated with BDI in the reward or loss domain in OCD and HVs; *w* was positively correlated with BDI in the loss domain only in HVs (Pearson *R*=0.303, *P*=0.039) and not in the reward domain or in OCD subjects (*P*>0.05).

There were no differences in *w* score between OCD subjects tested in the two sites in the reward (*P*=0.133) and loss domain (*P*=0.806).

The results of the behavioural analyses are reported in Supplementary Materials. The behavioural analyses qualitatively matched the modelling analyses, but several group effects failed to reach significance.

We also assessed whether the groups differed in tendency towards an action bias by assessing the probability of staying with the same stage 1 action as the previous stage 1 action (for example, left choice followed by left choice; *t*=0.223, *P*=0.823), or the probability of staying with the same stage 2 action as the previous stage 2 action (*t*=0.51, *P*=0.610), or the same (*t*=0.536, *P*=0.593) or different (*t*=0.66, *P*=0.507) stage 2 action as the previous stage 1 action.

#### Chronic antidepressant effects

As a proportion of the OCD subjects were on antidepressant medication, we then compared *w* in the reward condition in OCD subjects on (*N*=30; YBOCS 21.55 (s.d. 4.67); *w* 0.20 (s.d. 0.19)) and off (*N*=17; YBOCS 19.50 (s.d. 6.37); *w* 0.26 (s.d. 0.23)) chronic antidepressants (predominantly serotonin reuptake inhibitors) to HVs (*N*=90; 0.33 (s.d. 0.23)) using an ANOVA (F=5.072, *P*=0.007). Given the main group effect, we then compared the *post hoc* analysis: HVs had higher *w* or greater goal-directed learning relative to OCD subjects on selective serotonin reuptake inhibitors (SSRIs; *P*=0.003) but not off SSRIs (*P*=0.425).

**Figure 4:**
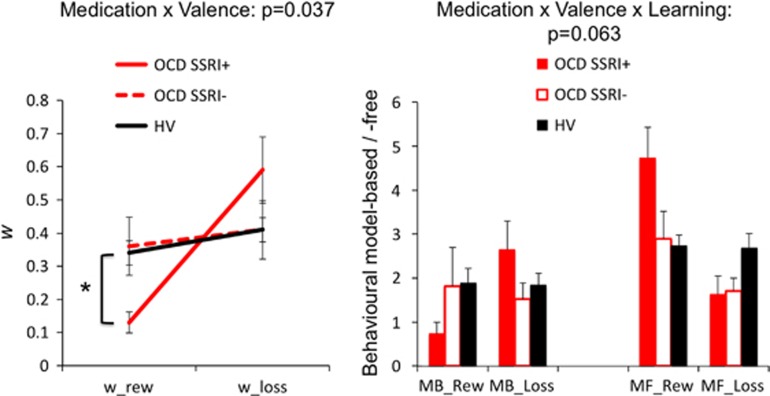
Effects of chronic SSRIs. The left graph shows the effects of chronic SSRIs on the computational analysis (*w*) and the right graph on the behavioural analyses. *P<0.005 on *post hoc* analysis for SSRI+ versus SSRI−. The healthy volunteer (HV) group is included in the graphs for the purposes of comparison. Error bars represent s.e.m. MB, model-based; MF, model-free; OCD, obsessive-compulsive disorder; Rew, reward; SSRI, selective serotonin reuptake inhibitor.

We also compared the influence of chronic SSRIs on *w* in both reward and loss outcomes in the OCD patients who had completed both tasks (Figure 4; SSRI+ *N*=16; SSRI− *N*=11). There were no group differences in age (SSRI+ 29.72 (s.d. 14.17); SSRI− 36.29 (s.d. 12.20), *P*=0.451), IQ (SSRI+ 114.62 (s.d. 6.91); SSRI− 117.58 (s.d. 4.79), *P*=0.211) or (YBOCS SSRI+ 21.55 (s.d. 4.67); SSRI− 19.50 (s.d. 6.37), *P*=0.274). There was an effect of valence (F(1,25)=7.322, *P*=0.012) and a medication × valence effect (F(1,25)=4.85, *P*=0.037). On *post hoc* analysis, OCD subjects on SSRIs had lower *w* scores in the reward condition (*P*=0.009) compared with off SSRIs with no difference in the loss condition (*P*=0.215). These findings confirm the findings in the larger group of OCD patients who had only completed the reward version. There were no main medication effects (F(1,25)=0.125, *P*=0.726). We further compared the behavioural goal-directed and habitual analysis and show a main effect of learning (F(1,25)=7.47, *P*=0.012), a valence × learning interaction (F(1,25)=7.78, *P*=0.010), with trend towards a valence effect (F(1,25)=3.806, *P*=0.063) and a medication status × valence × learning effect (F(1,25)=3.810, *P*=0.063; Figure 4).

#### Magnitude and order effects

**Figure 5:**
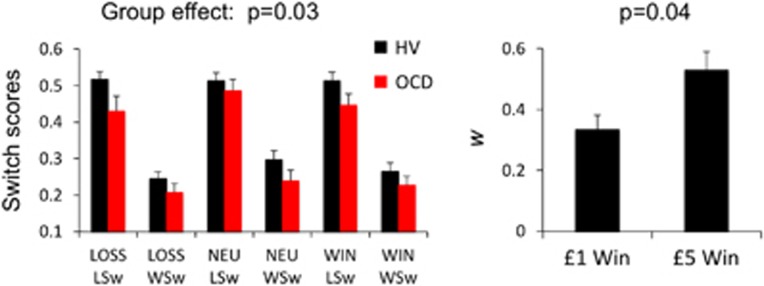
Switching and effects of reward magnitude. The left bar graph shows the switch scores for both lose-switch (LSw) and win-switch (WSw) in the one-step task for obsessive-compulsive disorder (OCD) and healthy volunteer (HV) subjects as a function of valence. The right bar graph shows the computational analysis for the two-step task in HVs as a function of low (£1) and high (£5) reward magnitude. Error bars represent s.e.m. NEU, neutral.

In the Supplementary Materials, we show that in two separate studies in HVs, greater reward magnitude (£5 versus £1) was associated with higher *w* scores (Figure 5) and that there were no effects of order in the modelling analysis.

#### OCD: one-step acquisition-reversal task

As the trials to criterion for acquisition and reversal were not normally distributed (Shapiro–Wilks *P*<0.0001), the non-parametric Mann–Whitney *U*-test was used to assess group differences (OCD: *N*=36; HV: *N*=72) focusing on the *a priori* hypothesis of loss acquisition and on an exploratory level, loss reversal. OCD subjects required fewer trials to reach criterion for the loss condition during acquisition (*P*=0.013) but not in the neutral (*P*=0.603) or reward condition (*P*=0.207) (Figure 2b). There were no significant differences during reversal in any condition (*P*>0.05).

We asked whether *w* and acquisition in the Loss condition was related in the two-step and one-step tasks, respectively (*N*=60 available data points). In the loss conditions, greater *w* learning (more goal-directed) was correlated with fewer trials to acquisition (better acquisition learning; Spearman rho=−0.355, *P*=0.005; Figure 2c) and with a trend towards more trials to reversal (worse reversal learning; 0.282, *P*=0.028).

We further assessed the lose-switch and win-stay scores (Figure 5). For the purposes of comparison with lose-switch, the win-stay scores were converted to win-switch scores (=1−win-stay). As the scores were normally distributed, using a mixed-measures ANOVA assessing a between-subjects factor of group (OCD, HV) and within-subjects factors of stay-switch (lose-switch, win-switch) and valence (reward, neutral, loss), there was a main group effect (F(1,88)=5.20, *P*=0.025), stay-switch effect (F(1,88)=173.606, *P*<0.0001) and a main valence effect (F(2,87)=3.249, *P*=0.043), and no interaction effects. Thus, OCD subjects were more likely to stay after both lose and win outcomes compared with HVs.

We also asked on an exploratory level whether OCD severity was associated with parameters in the loss acquisition phase including trials to acquisition, lose-switch or win-stay. YBOCS obsessive symptoms were positively correlated with lose-switch score (Pearson coefficient =0.463, *P*=0.012). There were no significant correlations between YBOCS obsessive symptoms and trials to acquisition or win-stay or YBOCS compulsive scores with other parameters (*P*>0.05).

## Discussion

We show a critical influence of outcome valence on the relative balance of goal-directed and habitual learning in OCD. In the context of reward outcomes, OCD is characterized by impaired goal-directed learning along with a relative shift towards enhanced habitual learning. For OCD patients (but not HVs), the pattern shifts with goal-directed learning enhanced and habitual learning impaired, relative to the reward condition. On separate analyses of the differential contribution of the two learning systems, we find that the effects of valence on OCD patients' behaviour are driven by differences in both goal-directed and habit learning between the two conditions, with the former increased and the latter decreased for loss relative to gain. We had previously published on this task in the reward condition in a smaller group of OCD patients.^6^ We now confirm this in a separate group of patients and further show that the two measures of goal-directed and habit learning are dissociable and both affected in OCD. There was also no relationship with action bias or the tendency to stay with the same side of choice as a function of the previous choice (for example, left side if previous choice left) at stage 1, 2 or during the transition.

Reactivity to losses in OCD was correlated between the two tasks, with greater goal-directed learning from loss outcomes in the two-step task correlated with greater acquisition from loss outcomes in the one-step task. We further show that OCD was associated with greater stay (or lower switch) behaviours irrespective of outcome valence but that obsession severity was positively correlated with a greater likelihood of switching after a loss outcome in the one-step task. Compulsion severity was positively correlated with habitual learning to reward outcomes in the two-step task. Our results highlight the clinical relevance for model-free learning to reward outcomes in OCD subjects and further suggest that OCD subjects may not be globally affected in goal-directed or habit learning but only in the context of sensitivity to rewards and losses. In HVs, *w* in the loss condition was positively correlated with depression scores suggesting that greater salience of negative outcomes with greater depression severity might enhance model-based goal-directed learning to losses. We thus emphasize that learning is influenced by motivational factors.

Our findings emphasize a role for valence effects. Given the known role of enhanced vigilance to aversive stimuli, our findings suggest that therapeutic approaches emphasize habituation of anxiogenic or aversive stimuli and its counterpart, enhancement of the salience of rewarding stimuli. The former is consistent with exposure therapy as the recommended psychological intervention of cognitive behavioural therapy (recommended by APA^23^ and Excellence NIfHaC^24^) in OCD, which utilizes exposure and response prevention to facilitate gradual habituation to anxiogenic stimuli. Our findings further emphasize a role for the latter, or enhancing the salience of rewarding stimuli that might steer cognitive resources away from salient losses and develop more flexible and goal-directed behaviours towards rewards.

### Goal-directed learning

Several studies have suggested impairments in action–outcome learning in OCD but in the context of correct or incorrect outcomes^11^ or reward versus no reward.^6^ However, these outcomes may be of less value in OCD subjects. We have recently shown that higher *w* or greater goal-directed behaviours in HVs correlate with greater volumes in medial orbitofrontal cortex and caudate.^6^ In contrast, in studies using implicit learning tasks which normally recruit striatal systems, OCD subjects have shown excessive activation in neural regions implicated in goal-directed systems such as the orbitofrontal cortex and medial temporal lobe.^9^ Although the former studies suggest impairments in the goal-directed system and an over-reliance on habit, the latter suggests an over-reliance on goal-directed systems. To resolve these disparate findings, an impairment in the capacity to arbitrate between model-free and model-based systems has been suggested.^25^

Alternatively, our findings suggest an important role for valence or motivational status. A generalized impairment in goal-directed learning would predict impairments across both valences. We confirm a shift in goal-directed learning in OCD subjects, with reduced model-based behaviour in the context of reward; however, we show higher model-based behaviour for losses relative to gains. Similarly, we show that OCD subjects have enhanced loss acquisition in the one-step task. Furthermore, both tasks correlate in the loss condition in OCD subjects, which suggests potentially analogous effects of loss outcomes. This may be related to greater sensitivity to loss outcomes as OCD subjects have been shown to use a negative learning bias; avoiding stimuli associated with negative outcomes maintained by faster responses and higher rates of aversive avoidance compared with HVs.^13^ Alternatively this may represent enhanced learning of negative values over all the trials, a phenomenon associated with obsessions but not anxiety,^13, 17^ suggesting that harm avoidance may be an independent contributor. These findings also suggest that rather than an impairment in goal-directed learning *per se*, the findings may be specific to motivational status.

We further show that in HVs, greater reward magnitude or salience is associated with a shift towards greater goal-directed behaviours. These findings are compatible with the concept that the shifts observed in OCD are a function of outcome salience.

### Habitual learning

We show that OCD is associated with a specific enhancement in habitual learning to reward outcomes and that this enhanced habitual learning correlates with greater YBOCS compulsivity severity scores. By testing both reward and loss outcomes simultaneously, our results suggest that compulsive severity is related to a primary abnormality of enhanced habitual learning as a function of positive reinforcement.

Although OCD subjects have shown enhanced habit expression to shock outcomes,^17^ we did not observe any difference in habitual learning to loss outcomes. We did not observe any differences in habitual learning to loss outcomes, which may be related to different measures assessed by the tasks. The shock avoidance task involves over-training of action–outcome contingencies followed by testing after outcome devaluation. The task also uses shock outcomes, which may be more physically motivating, whereas the two-step task uses monetary outcomes. Relief from painful stimuli may differ from the avoidance of loss stimuli. The two-step task tests relative differences in the two systems of learning presuming an opposing relationship and focuses on an earlier stage of learning rather than after over-training. Direct comparison of the two tasks has indicated that preference for a valued rather than devalued, over-trained stimulus (indicating goal-directed choice) correlates with model-based but not model-free learning in the current two-step task;^26^ however, this comparison spanned only appetitive food items and monetary reward, respectively.

### Chronic SSRIs

We show that OCD subjects on chronic SSRIs have lower goal-directed behaviours to reward but greater goal-directed behaviours to losses compared with those not on SSRIs. These findings are driven particularly by the reward condition. Our findings suggest that the decrease in goal-directed behaviours in OCD subjects in the reward condition of the two-step task may be driven by those on chronic SSRIs rather than untreated subjects.

Convergent evidence suggests OCD is characterized by abnormalities in serotonergic function. Multiple randomized controlled trial studies show that OCD subjects respond to SSRIs.^27^ Drug-naive OCD subjects further show lower serotonin transporter availability as measured using [^11^C]DASB positron emission tomography imaging in the thalamus and midbrain,^28^ insula,^29^ amygdala, anterior cingulate, nucleus accumbens and striatum.^30^ Using 123I-BetaCIT serotonin transporter ligand single-photon emission computed tomography study, both decreased midbrain–pons and thalamus–hypothalamus^31, 32, 33, 34^ binding was observed in OCD subjects although one study reported increased^35^ binding in the midbrain–pons. However, using [^11^C]McN 5652 serotonin transporter radiotracer, no differences were observed between OCD and HVs.^36^ The role of the 5HT(2A) receptor availability is less clear with a study reporting decreased binding in frontopolar, dorsolateral, medial frontal and parietal regions in OCD using [^11^C]MDL,^37^ whereas another study reported increased caudate binding using [^18^F]altanserin, which normalized with SSRIs.^38^ Together, these studies suggest abnormalities in serotonergic function in OCD.

These findings stand in contrast to a recent study in HVs in which acute tryptophan depletion, which decreases central serotonin levels, impairs goal-directed learning to rewards and enhances goal-directed learning to losses, an effect we suggest to be related to the influence on average reward representation.^39^ The effects of chronic SSRIs in OCD similarly decreases goal-directed behaviours in the reward condition relative to those not on SSRIs. However, one might anticipate that if chronic SSRIs simply represented the opposite of tryptophan depletion with enhanced serotonin levels, then this finding is inconsistent with our recent findings in HVs. These findings also suggest that chronic SSRIs may be driving the differences between OCD subjects and controls. However, although there were no differences in demographics or severity of OCD, the OCD patients on SSRIs may initially have had more severe OCD symptoms and hence greater serotonergic abnormalities with improvement of symptoms following antidepressant use. This inconsistency may thus reflect either underlying differences in serotonergic tone in OCD subjects who are on SSRIs versus those who are not or may reflect the complexity of chronic SSRIs effects, which might have an influence on serotonin receptor density rather than only a simple effect on serotonin levels. We note that side effects of high doses of SRRIs on concentration or sleepiness may have had some influence on performance thus shifting away from model-based strategies although we might expect this to be observed across both reward and loss domains.

### Stay-switch

On the one-step task, OCD subjects had an overall greater likelihood of staying with the same chosen stimulus rather than switching compared with HVs. This effect was irrespective of the outcome, indicating generalized perseveration or decreased switching. Impairments in behavioural flexibility and shifting, particularly attentional shifting, have been suggested in OCD,^40, 41, 42^ but this cognitive measure implicates a much higher-order attentional mechanism than perseveration. The current findings pose some therapeutic relevance, providing directionality to behavioural therapy with a suggestion that encouragement of more switching and sampling behaviours may be favourable.

We further showed that YBOCS obsessionality severity scores correlated with a greater loss-switch rate. This suggests a relationship between obsessions and an automatic avoidance response. Thus, obsession severity and an avoidance response to an aversive outcome may reflect a separable underlying stimulus-response learning mechanism dissociable from either goal-directed or habitual learning.

### Limitations

The reversal task may be complicated by a lack of distinct separation of reward and loss outcomes along with differences in the average reward or punishment values. A design with a more clear separation of reward and loss outcomes may be indicated. However, we show specificity to the loss condition and further, a correlation with the loss condition of the two-step task. This suggests a specificity of the loss condition in the one-step task towards loss outcomes. OCD subjects were also tested in the reward then loss condition of the two-step task suggesting a possible role for an order effect to which OCD subjects may be more susceptible. That the study was conducted across 2 sites is not ideal but provides some presentation of generalizability of the results given that there were no significant site effects. An order effect was ruled out in HVs but not in OCD subjects. OCD subjects may be better at learning and generalization between tasks; however, the same order of testing was conducted in both OCD subjects and HVs.

## Conclusion

We highlight the influence of motivational processes on goal-directed and habitual behaviours in OCD. Although the role of the serotonergic system remain elusive, future studies should further examine the effects of SSRIs on reward processing and its influence on habitual or goal-directed behaviours in this population.

## Figures and Tables

**Table 1:** Characteristics of subjects and parameters of two-step task

			*OCD*	*HV*	*T or Chi-square*	P-*value*
Age			39.46 (13.89)	38.53 (14.11)	0.29	0.77
Gender			20F: 10M	35F: 18M	0.003	0.95
IQ			115.87 (6.32)	117.06 (5.89)	0.86	0.39
BDI			14.34 (9.30)	4.03 (4.76)	−6.23	<0.0001
YBOCS total			20.81 (5.45)			
YBOCS compulsion			10.05 (3.31)			
YBOCS obsession			10.76 (3.11)			

Reward	B_1_	Choice randomness	4.96 (3.83)	4.56 (2.80)	−0.61	0.54
	B_2_	Choice randomness	2.78 (1.84)	3.59 (1.74)	2.28	0.03
	L_1_	Learning rate	0.46 (0.31)	0.50 (0.28)	0.73	0.47
	L_2_	Learning rate	0.41 (0.32)	0.42 (0.26)	0.11	0.91
	Lambda	Reinforcement eligibility	0.49 (0.34)	0.63 (0.25)	2.42	0.02
	P	Perseveration	0.22 (0.21)	0.21 (0.17)	−0.20	0.84
	LL	Negative log likelihood	213.84 (45.48)	208.35 (37.69)	−0.59	0.55
						
Loss	B_1_	Choice randomness	4.55 (3.28)	4.50 (3.37)	−0.06	0.95
	B_2_	Choice randomness	1.44 (1.37)	2.56 (1.54)	3.42	0.001
	L_1_	Learning rate	0.42 (0.27)	0.52 (0.31)	1.58	0.12
	L_2_	Learning rate	0.35 (0.37)	0.52 (0.24)	2.72	0.008
	Lambda	Reinforcement eligibility	0.41 (0.34)	0.52 (0.32)	1.58	0.12
	P	Perseveration	0.42 (0.32)	0.29 (0.28)	−2.00	0.05
	LL	Negative log likelihood	210.56 (37.45)	211.35 (47.90)	0.07	0.94

Abbreviations: BDI, Beck Depression Inventory; F, female; HV, healthy volunteer; M, male; OCD, obsessive-compulsive disorder; YBOCS, Yale Brown Obsessive Compulsive Scale.
