# Supplementary figures and images for: Motivation and value influences in the relative balance of goal-directed and habitual behaviours in obsessive-compulsive disorder
Source: Transl Psychiatry. 2015 Nov 3;5(11):e670–. doi: 10.1038/tp.2015.165 (PMC5068758; doi:10.1038/tp.2015.165)

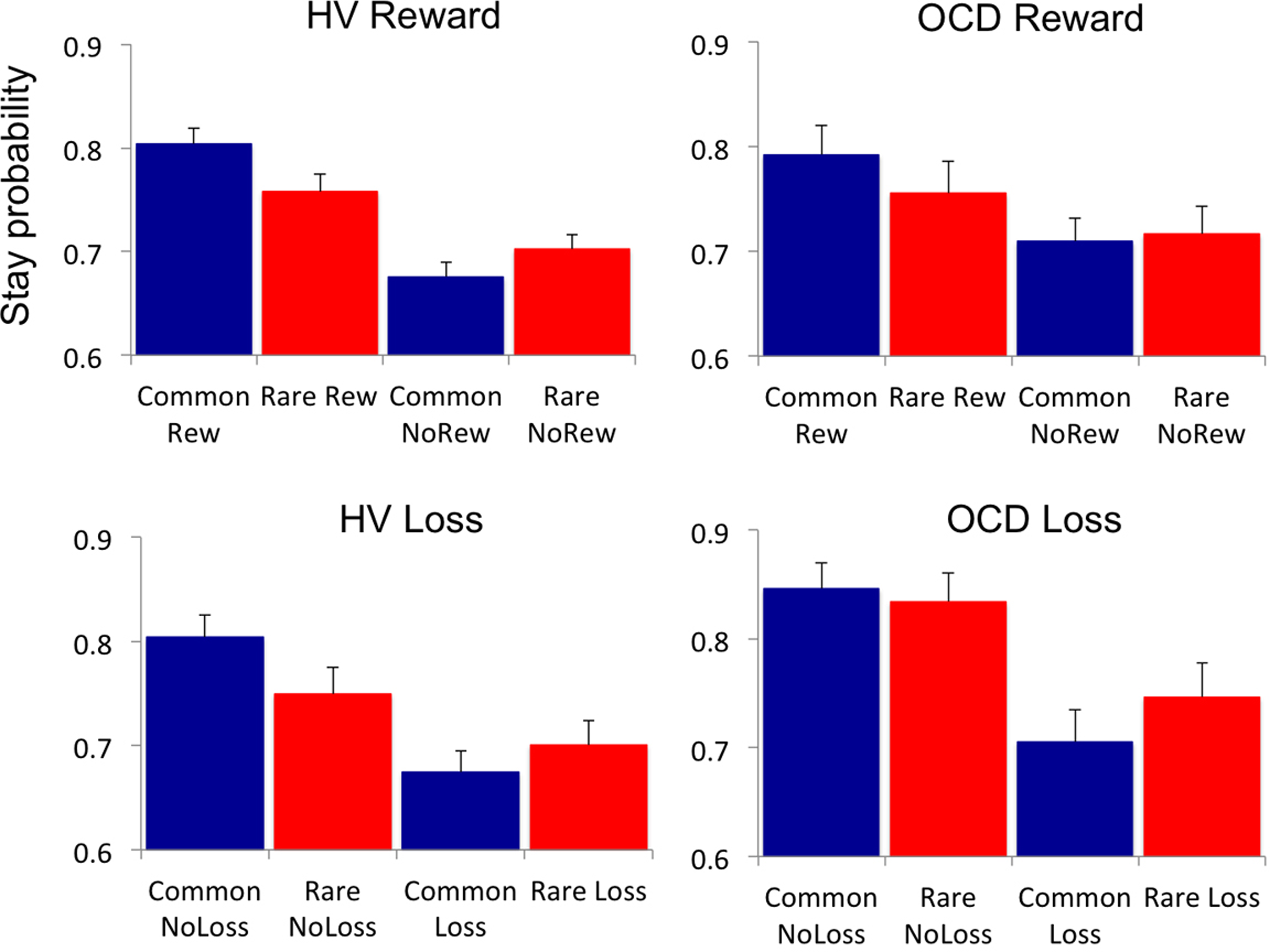

Supplement: Supplementary Figure 1 [file tp2015165x1.tif]

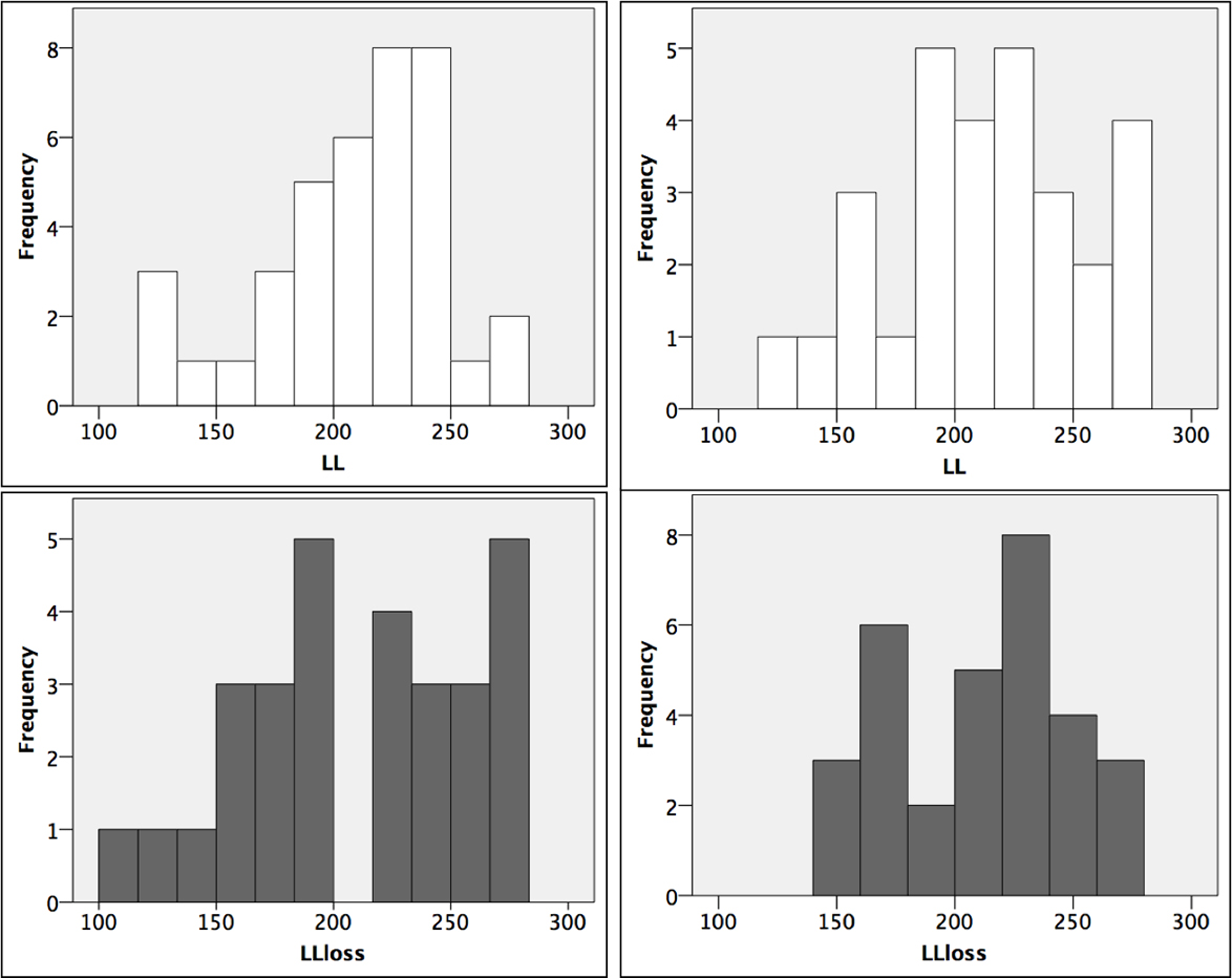

Supplement: Supplementary Figure 2 [file tp2015165x2.tif]
