# Supplementary material for: Motivation and value influences in the relative balance of goal-directed and habitual behaviours in obsessive-compulsive disorder
Source: Transl Psychiatry. 2015 Nov 3;5(11):e670–. doi: 10.1038/tp.2015.165 (PMC5068758; doi:10.1038/tp.2015.165)
Supplement: Supplementary Information [file tp2015165x3.pdf]

Model-free temporal difference algorithm (habit)

The SARSA (λ) temporal difference (TD) algorithm was used to model the habitual strategy. Choices are based on previously reinforced actions in which the reinforcer is equivalent to the TD reward prediction error (RPE, δ). At each stage *i* of each trial *t*, TD action values (Q_{TD}) are determined for each state-action pair (a_i) for each state (s_i) at Stage 1 (s_A) and the two states at Stage 2 (s_B and s_C) (Figure 1).

$$Q_{TD}(s_{i,t},a_{i,t}) = Q_{TD}(s_{i,t},a_{i,t}) + \alpha_i \lambda \delta_{i,t}$$

where

$$\delta_{i,t} = r_{i,t} + Q_{TD}(s_{i+1,t},a_{i+1,t}) - Q_{TD}(s_{i,t},a_{i,t})$$

The action values (Q_{TD}) are updated based on the RPE (δ). For the immediately rewarded Stage 2, the current action value is modified by the reward (r = £1 or £0) and $Q_{TD}(s_{i+1,t},a_{i+1,t}) = 0$. For Stage 1, the RPE is modified by the updated Stage 2 action values $Q_{TD}(s_{i+1,t},a_{i+1,t})$ and r=0. A separate learning rate (α) is calculated for each stage (α_1, α_2). The reinforcement eligibility parameter (λ) determines the extent to which the final outcome (λ = 1) or the Stage 2 action values (λ = 0) are used as a model-free reinforcer for the Stage 1 action value.

Model-based reinforcement learning algorithm (goal-directed)

The model-based reinforcement-learning algorithm was computed by mapping state-action pairs to a transition function and assuming two possible choices: common transition: $P(S_B \mid S_A, a_A) = 0.7$, $P(S_C \mid S_A, a_B) = 0.7$ or rare transition: $P(S_B \mid S_A, a_A) = 0.3$ $P(S_C \mid S_A, a_B) = 0.3$ where a is one of two actions (a_A or a_B) The model-based reinforcement learning algorithm calculated the Stage 1 action value (Q_{MB}) for each trial based on the probabilities of each state and the maximum action value of that state over all trials $\max Q_{TD}(s_i,a)$.

$$Q_{MB}(s_A, a_j) = P(s_B | s_A, a_{i,}) \max Q_{TD}(s_B, a) + P(s_C | s_A, a_{i,}) \max Q_{TD}(s_C, a)$$

26

The weighted sum of the model-free and model-based values was then calculated for the first stage.

$$Q_{net}(s_A, a_j) = w\, Q_{MB}(s_A, a_j) + (1-w)\, Q_{TD}(s_A, a_j)$$

where w is the weighting parameter in which w=0 indicates a reliance on model-free (habit) strategies and w=1 indicates a reliance on model-based (goal-directed) strategies. At the second stage, $Q_{net} = Q_{MB} = Q_{TD}$.

The probability of a choice was calculated using the softmax equation for Q_{net}:

$$P(a_{i,t} = a | s_{i,t}) = \frac{\exp(\beta_i [Q_{net}(s_{i,t}, a) + p * \mathrm{rep}(a)])}{\Sigma_{a'} \exp(\beta_i [Q_{net}(s_{i,t}, a) + p * \mathrm{rep}(a)])}$$

The inverse free parameter β_i is an index of choice reliability calculated for both stages (β_1, β_2) with a lower value indicating greater choice randomness. p is an index of perseveration ($p>0$) or switching ($p<0$) in the first stage choices.

We also compared model-based and model-free reinforcement learning separately rather than their relative weighting by computing: model-based = β * w and model-free = $\beta * (1 - w)$.

Two-step task: behavioural analysis:

Model-based or model-free learning are predicted to produce different stay or switch patterns at Stage 1 based on the influence of the transition probability (common or rare) and outcome (reward or no reward) of the previous trial. For instance, model-free learning predicts only a main effect of outcome whereas model-based learning is an interaction of outcome by transition probability. Previous studies on healthy volunteers have shown an intermediate pattern (i.e. using both model-based and model-free strategies) of choice preference suggesting parallel use of both strategies (Daw *et al.*, 2011).

Results

Computational analyses

The model fits (log-likelihoods) were all normally distributed in both OCD and healthy volunteer groups (Shapiro-Wilks: $p>0.05$). Histogram plots of the log-likelihoods are plotted in Figure S2.

Valerie Voon 31/7/2015 13:52
Formatted: Font:Italic

Valerie Voon 31/7/2015 13:52
Formatted: Font:(Default) Times New Roman

Behavioural analyses

We further performed a behavioural analysis of the frequency of switching at Stage 1 as a function of the previous trial's events. We analyzed the HV and OCD groups separately then compared the two groups. We analyzed the within subjects factor of Outcome (Reward or No Reward) and Transition (common or rare) (Figure S1).

Reward: In the HV, there was a main effect of Outcome ($F(1,95)=62.08$, $p<0.0001$) suggesting a greater likelihood of stay after a reward and a Transition x Outcome interaction ($F(1,95)=18.61$, $p<0.0001$) indicating parallel use of goal-directed and habitual behaviours (Figure 3). In the OCD group, there was a main effect of Outcome ($F(1,46)=10.79$, $p=0.002$) and no interaction between Transition x Outcome interaction ($F(1,46)=3.157$, $p=0.082$) indicating predominant reliance on habit learning. However, there was no interaction with Group in the interactions: Group x Outcome ($F(1,142)=2.187$, $p=0.142$) or Group x Outcome x Transition ($F(1,142)=0.983$, $p=0.323$).

Loss: In both HV and OCD subjects, as expected there was a main effect of Outcome (HV: $F(1,44)=31.48$, $p<0.0001$); OCD: $F(1,31)=19.68$, $p<0.0001$) and a Transition x Outcome interaction (HV: $F(1,41)=10.89$, $p=0.002$; OCD: $F(1,31)=4.711$, $p=0.038$). However, there was no interaction with Group in the interactions: Group x Outcome ($F(1,74)=0.860$, $p=0.357$) or Group x Outcome x Transition ($F(1,74)=0.595$, $p=0.443$).

Computational analysis of second site

Valerie Voon 31/7/2015 13:52
Formatted: Font:Italic

We further analyzed the group tested at the Karolinska separately using a mixed measures ANOVA (reward w: HV: 0.34 (SD 0.28), OCD 0.15 (SD 0.12); loss w: HV: 0.35 (SD 0.30), OCD 0.48 (0.39). We show a main effect of Valence (p=0.026) and a Group by Valence interaction (p=0.035). Posthoc analyses show that the OCD subjects have lower w scores in the reward condition compared to healthy volunteers (p=0.026) with no group differences in the loss condition (p=0.338).

Valerie Voon 31/7/2015 13:52
Formatted: Font:(Default) Times New Roman

Order effects

In the 53 HV tested with £1 Reward then £1 Loss, there was no effect of Valence (N=32; F(1,31)=1.215, p=0.299). To ensure there was no order effect of testing, the 53 HV tested with £1 Reward (*w*=0.34 (SD 0.25)) then £1 Loss (*w*=0.42 (SD 0.25)) were then compared with an additional 15 HV tested with £1 Loss (*w*=0.35 (SD =0.30)) followed by £1 Reward (*w*=0.29 (SD 0.22)). There was no Order effect (F(1,66)=1.083, P=0.302, η^2=0.01) or Outcome effect (F(1,66)=1.720) p=0.194, η^2=0.01) or interaction between Order and Outcome (F(1,66)=0.037, p=0.848, η^2=0.002).

Valerie Voon 31/7/2015 13:52
Formatted: Superscript

Reward magnitude or salience effect

To further understand the shifts between the relative balance of goal-directed and habitual behaviours and assess the influence of reward magnitude or salience, we compared a new set of 20 HV tested in a within-subject randomized order with either £1 Reward (*w* 0.35 (SD 0.29)) or £5 Reward (*w* 0.53 (SD 0.28)). The £5 reward was associated with higher *w* relative to the £1 reward (F(1,19)=5.124, p=0.036) suggesting that greater reward magnitude or salience was associated with greater goal-directed behaviours (Figure 5).

Valerie Voon 31/7/2015 13:52
Formatted: Font:Italic

Valerie Voon 31/7/2015 13:52
Formatted: Font:Italic

Valerie Voon 31/7/2015 13:52
Formatted: Font:Italic

One-step acquisition task

To assess the effects of chronic antidepressant use, we further compared trials to criterion for acquisition for Loss between healthy volunteers (N=60; 8.36 (SD 6.37)) and OCD subjects on (N=15; 4.53, (SD 4.49)) and off (N=12; 4.08 (SD 5.88)) antidepressants using the Mann-Whitney U test. As there was a significant group difference (p=0.001), we then examined post-hoc differences: OCD subjects both on (p=0.009) and off (p=0.015) had a lower number of trials to criterion relative to healthy volunteers with no difference between on and off antidepressants (p=0.399). On an exploratory analysis, we also compared trials to criterion for acquisition for Reward; there were no significant group differences (p=0.408).

Relationship between stay behaviours (perseveration) on the one-step task and two-step outcomes

On an exploratory basis, we examined the relationship between perseveration or the tendency to stay irrespective of outcome (for the win and loss conditions) on the one-step task and the outcome parameters of the two-step task. We show that across all subjects driven by the healthy volunteers, w was negatively correlated with win-switch (reported as Pearson correlation coefficient, p-value: All subjects: -0.219, p=0.018; HV: -0.244, p=0.023; OCD: -0.169, p=0.364) and positively correlated with lose-switch (All subjects: 0.194, p=0.036; HV: 0.185, p=0.089; OCD: 0.121, p=0.517) but only in the loss condition and not in the win condition (p>0.05). Similarly, both the computationally derived (All subjects: -0.280, p=0.002; HV: -

Valerie Voon 31/7/2015 13:52
Formatted: Font:(Default) Times New Roman

Valerie Voon 31/7/2015 13:55
Formatted: Line spacing: double

Valerie Voon 26/7/2015 18:37
Moved down [1]: *Relationship between stay behaviours (perseveration) on the one-step task and two-step outcomes*

Valerie Voon 26/7/2015 18:37
Moved (insertion) [1]

Valerie Voon 31/7/2015 13:52
Formatted: Font:(Default) Times New Roman

Valerie Voon 31/7/2015 13:55
Formatted: Line spacing: double

0.338, p=0.002; OCD: -0.059, p=0.754) and behavioural model-based (All subjects: -0.209, p=0.033; HV: -0.235, p=0.045; OCD: -0.143, p=0.444) measures were negatively correlated with win-switch in the loss condition but not with lose-switch or in the win condition (all other p>0.05). Thus, w is unrelated to general perseveration but higher w or goal-directed behaviours on the two-step task are related to the tendency to adaptively switch or stay following outcomes on the one-step task following a win and stay. These findings are relevant only in healthy volunteers and in the one-step loss conditions.

Legend

Figure S1. Stay probability for reward and loss

Figure S2. Histogram plots of model fits.

The histogram plots show the log-likelihood of the reward (top) and loss conditions (bottom) in the healthy volunteers (left) and OCD subjects (right).

Daw ND, Gershman SJ, Seymour B, Dayan P, Dolan RJ (2011). Model-based influences on humans' choices and striatal prediction errors. *Neuron* **69:** 1204-1215.

Valerie Voon 26/7/2015 18:37

Deleted: On an exploratory basis, we examined the relationship between perseveration or the tendency to stay irrespective of outcome (for the win and loss conditions) on the one-step task and the outcome parameters of the two-step task. We show that across all subjects driven by the healthy volunteers, w was negatively correlated with win-switch (reported as Pearson correlation coefficient, p-value: All subjects: -0.219, p=0.018; HV: -0.244, p=0.023; OCD: -0.169, p=0.364) and positively correlated with lose-switch (All subjects: 0.194, p=0.036; HV: 0.185, p=0.089; OCD: 0.121, p=0.517) but only in the loss condition and not in the win condition (p>0.05). Similarly, both the computationally derived (All subjects: -0.280, p=0.002; HV: -0.338, p=0.002; OCD: -0.059, p=0.754) and behavioural model-based (All subjects: -0.209, p=0.033; HV: -0.235, p=0.045; OCD: -0.143, p=0.444) measures were negatively correlated with win-switch in the loss condition but not with lose-switch or in the win condition (all other p>0.05). Thus, w is unrelated to general perseveration but higher w or goal-directed behaviours on the two-step task are related to the tendency to adaptively switch or stay following outcomes on the one-step task following a win and stay. These findings are relevant only in healthy volunteers and in the one-step loss conditions.”

Valerie Voon 31/7/2015 13:52

Formatted: Font:(Default) Times New Roman

Valerie Voon 31/7/2015 13:52

Formatted: Font:(Default) Times New Roman

Valerie Voon 31/7/2015 13:52

Formatted: Font:(Default) Times New Roman
